# Sleep Characteristics Among Children with a Parental History of Alcohol Use Disorder

**DOI:** 10.1007/s40429-024-00602-x

**Published:** 2024-10-05

**Authors:** Maria M. Wong, Madisen Hillebrant-Openshaw

**Affiliations:** 1 Department of Psychology, Idaho State University, Pocatello, ID 83209-8112 USA; 2 Department of Psychology and Neuroscience, University of Colorado Boulder, Boulder, CO 80309 USA

**Keywords:** Sleep, Children, Adolescents, Parental history of alcohol use disorder

## Abstract

**Purpose of Review:**

The purpose of the review was to examine findings on sleep characteristics among children with a parental history of alcohol use disorder (CPHAUDs) in different age groups. We identified unanswered questions and discussed directions for future research. We also discussed the implications of these current findings on alcohol prevention and intervention programs.

**Recent Findings:**

Parental ratings and youth report of sleep difficulties have been longitudinally associated with the emergence of alcohol use and alcohol-related problems among both CPHAUDsand non-CPHAUDs. There were inconsistent findings comparing sleep characteristics in these two groups. Studies that used self-report and parental ratings reported no or minimal differences while studies that used actigraphy and polysomnography found significant, albeit moderate but meaningful differences.

**Summary:**

Current research shows that CPHAUDs and non-CPHAUDs are similar on most objective and subjective sleep measures. There are a few significant differences between the two groups that may have implications for the development of behavioral problems, substance use and other risk behaviors.

## Introduction

According to a report by the Substance Abuse and Mental Health Administration (SAMHSA) in 2017, approximately 10.5% (7.5 million) of U.S. children 17 years old or younger live with a parent with alcohol use disorder [1, 2]. Children with a parental history of alcohol use disorder (CPHAUDs) are at a higher risk of early onset of alcohol use and other substance use compared with non-CPHAUDs [3–5]. They are also more likely to develop alcohol related problems (e.g., binge drinking, driving under the influence of alcohol), alcohol use disorder (AUD) and substance use disorders (SUD) [6–8]. However, the mechanisms that may explain the increased risk of alcohol and other substance use among CPHAUDs are not fully understood.

Sleep disturbances including insomnia symptoms and short sleep duration have been longitudinally linked to early onset of substance use and related problems. Is the higher risk of substance use and problems among CPHAUDs related to sleep disturbances or other sleep parameters? In the sections below, we reviewed studies comparing CPHAUDs and non-CPHAUDs on subjective (e.g., parental ratings of sleep, self-report) and objective sleep measures (i.e., actigraphy, polysomnography). We discussed the implications of current research findings on prevention and intervention programs. In the last section, we identified questions that still need to be addressed and provided suggestions for future research.

## Methods

Studies were identified using OneSearch, PubMed, PsycINFO, PsycARTICLES, and Google Scholar. Searches were conducted between December 13th and February 9th 2023. Search terms in the title and abstract included: sleep OR sleep problems, sleep deprivation OR sleep disturbance OR sleep quality OR insomnia AND children of alcoholics OR children of alcoholic parents OR parental drinking OR parent history of alcoholism OR parent problem drinking OR family history of alcoholism OR parental substance use. Additional criteria for searches included academic articles that were peer-reviewed. We did not include an earliest possible date in the search. A total of 490 articles resulted from these search criteria. From these results, 16 articles were included and 474 were excluded. Reasons for exclusion included lack of parental substance use information, lack of information about participant sleep, focus on prenatal alcohol exposure/fetal alcohol spectrum disorder, or lack of empirical study and results. A list of included articles is shown in Table 1.

**Table 1:** Summary of research findings

Study	Sample	CPHAUDs	Age	Gender	Race/Ethnicity	Study Findings
**Parental Alcohol Use Disorder Diagnosis**						
Conroy et al., 2015	*N* = 92 Children and their parents	74% COA 26% non-COA	7.2–12.9 years [*M* = 10.2(1.2)]	37% F 63% M	55% White	CPHAUDs were more likely to sleep for a shorter duration at night, to take more naps, and move more during sleep as shown in both self-reports and actigraphy.
Dahl et al., 2003	*N* = 32 Children and Adolescents	56% COA 44% non-COA	8–16 years	53% F 47% M	90.6% White	Male CPHAUDs showed differences in microstructure sleep compared to non-CPHAUDs. No differences were found in females. All participants were clinically depressed.
Hariston et al., 2016	*N* = 82 Children	79% COA 21% non-COA	7.2–13.0 years [*M* = 10.5(1.3)]	35% F 65% M	52% White 18% Black 13% Hispanic 16% Not listed	CPHAUDs had shorter total sleep times then non-CPHAUDs. CPHAUDs also went to bed later and spent less time in bed on weekends compared to non-CPHAUDs.
Tarokh & Carskadon, 2010	*N* = 30 Children	43% COA 57% non-COA	9–10 years	37% F 63% M	Not reported	Sleep stages did not differ between CPHAUDs and non-CPHAUDs. CPHAUDs had less normalized power in the delta band and spindle range during NREM compared to non-CPHAUDs.
Tarokh et al., 2012	*n =* 24 Children and *n =* 25 Adolescents	Children: 42% CPHAUDs 58% non-COA Teens: 40% CPHAUDs 60% non-COA	Children: 9–10 years Adolescents: 15–16 years	Children: 33% F 67% M Teens: 64% F 36% M	Not reported	In both the child and teen samples, CPHAUDs and non-CPHAUDs were not different on any sleep stage variables. In the child sample, CPHAUDs showed lower power in the sigma band in the initial assessment. In the teen sample, CPHAUDs showed lower NREM sleep EEG power in the delta band at both assessments.
Wong et al., 2004	*N* = 258 Children and their parents	60% COA 40% non-COA	3–5 years old at T1	0% F 100% M	100% White	Sleep problems in children predicted early use of substances (alcohol, marijuana, illicit drugs, cigarettes) in adolescence, but no differences on sleep measures were found between CPHAUDs and non-CPHAUDs.
Wong et al., 2009	*N* = 386 Children and their parents	75% COA 25% non-COA	3–5 years old at T1	24% F 76% M	100% White	Sleep problems in children predicted early use of alcohol, marijuana and cigarettes in boys, and alcohol in girls. CPHAUDs and non-CPHAUDs were not different on sleep variables.
Wong et al., 2010	*N* = 386 Children and their parents	75% COA 25% non-COA	3–5 years old at T1	24% F 76% M	100% White	Maternal ratings of sleep problems in childhood predicted self-report of sleep problems in adolescence, which in turn was associated with lower response inhibition in adolescence and earlier onset of alcohol-related problems in young adulthood. CPHAUDs and non-CPHAUDs were not different on sleep variables.
Wong et al., 2018	*N* = 115 Children and their parents	67% COA 33% non-COA	8–12 years [*M* = 10.9(1.5)]	56% F 44% M	76% White 11% Hispanic 12% Others	CPHAUDs were more likely to be rated as overtired by their parents compared to non CPHAUDs. No differences on other self-reports or parent-reports of sleep were found.
Wong et al., 2022	*N* = 248 Children and their parents	48% COA 52% non-COA	8–12 years [*M* = 10.4(1.4)]	50% F 50% M	64% White 28% Hispanic 8% Others	CPHAUDs were more likely to report sleeping in a poor environment and had an unstable sleep schedule than non CPHAUDs. They were more likely to have a shorter sleep duration (actigraphy) than non-CPHAUDs and a longer sleep onset latency (PSG) compared with non-CPHAUDs.
Wong et al., 2023	*N* = 248 Children and their parents	48% COA 52% non-COA	8–12 years [*M* = 10.4(1.4)]	50% F 50% M	64% White 28% Hispanic 8% Others	CPHAUDs were more likely to report sleeping in a poor environment, which is associated with the development of internalizing and externalizing problems over time.
**Parental Alcohol Use**						
Kelly & El-Sheikh., 2016	*N* = 282 Children and their parents	3% Mothers and 12% Fathers with problem drinking	8–12 years [*M* = 9.4(8.6)]	48% F 52% M	65% White 35% Black	Parent problem drinking did not directly predict sleep duration, sleep efficiency, or long wake episodes across all participants. However, these relationships were significant among Black and low SES participants.
Kelly & El-Sheikh., 2019	*N* = 280 Children and their parents	5% Mothers and 9% Fathers with problem drinking	*M* = 10.3(8.1) years	45% F 55% M	66% White 34% Black	Father problem drinking predicted reduced sleep duration and efficiency in children over time. Further, more frequent long wake episodes in children predicted more parental problem drinking.
Lund et al., 2023	*N* = 8773 Adolescents	6.8% Children with binge drinking in both parents	*M* = 16.1(1.8) years	50% F 50% M	Not reported. Study Conducted in Norway	Parent weekly binge drinking, and frequent heavy drinking predicted a higher likelihood of adolescent children being prescribed a medication for sleep.
**Family History of Alcohol or Substance Use**						
Arnedt et al., 2011	*N* = 93 Young adults	31% COA 69% non-COA	21–31 years [*M* = 24.4(2.7)]	63% F 37% M	82% White 3% Black 4% Asian 11% Others	Family history of alcoholism was related to less sleepiness and higher sleep quality compared to those without a family history of alcoholism after alcohol administration. The authors suggested that one has to interpret the results with caution as their sample size was small. Otherwise, no differences were found.
Lees et al., 2021	*N* = 11,873 Children	26% COA 74% non-COA	9.0–10.9 years [*M* = 9.9(0.6)]	48% F 52% M	51% White 15% Black 13% Hispanic 2% Asian 11% Others	Children with a family history of substance misuse had more sleep disturbances than children without this history.

## Parental History of AUD and Offspring Sleep Characteristics

### Prenatal Exposure to Alcohol

In considering the effects of parental history of alcohol use disorder on children’s sleep, we did not include studies on individuals with prenatal exposure to alcohol. Prenatal alcohol exposure may result in fetal alcohol spectrum disorders (FASD), which affects approximately 1 to 5% of the population in the U.S [9–11]. Fetal alcohol syndrome (FAS) is the most severe disorder on the spectrum. FASD have adverse consequences on children’s physical characteristics, neurocognitive abilities and behaviors, including sleep. Children with FASD have reduced total sleep time, more frequent awakenings and disrupted sleep patterns compared to children without FASD. The results are consistent across studies using different measures of sleep, including polysomnography (sleep electroencephalography), actigraphy and subjective reports. CPHAUDs with FASD likely have different etiological pathways of alcohol and mental health problems compared to other CPHAUDs. Due to this reason, we did not include research on FASD children in this paper. A thorough review on prenatal exposure to alcohol and sleep can be found in [12]. A discussion of the epidemiology, diagnosis of FASD and associated neurocognitive and behavioral deficits can be found in [13, 14].

### Subjective Measures of Sleep

There are many validated questionnaires to measure sleep in pediatrics populations [15–17]. These questionnaires were designed to gather parental ratings or youth report of different aspects of sleep. Examples include the Pediatrics Sleep Questionnaire (parental ratings) [18], Children’s Report of Sleep Patterns (youth report) [19–21], and Children’s Sleep Habits Questionnaire (youth report) [22].

#### Parental Ratings

In studies that link poor sleep with subsequent early onset of alcohol use, CPHAUDs and non-CPHAUDs did not differ on maternal ratings of having trouble sleeping and overtiredness, as measured by the Child Behavior Checklist (CBCL) [23]. The two groups were similar on these ratings in both early (*N* = 258; 3–5 years old; 60% CPHAUDs; 0% girls) [24] and middle childhood (*N* = 386; 3 to 8 years old; 75% CPHAUDs; 24% girls) [25].

One study compared CPHAUDs and non-CPHAUDs on multiple sleep measures (*N* = 115; *M*_age_=10.85 (*±* 1.51); 8 to 12 years old; 67% CPHAUDs; 56% girls) [26]. Data were drawn from Time 1 of a three-year longitudinal study. Controlling for age, sex and ethnicity, the two groups did not differ on parental ratings of most of the CBCL sleep items, including “nightmares”, “sleeps less than most kids”, “sleeps more than most kids during day and/or night”, “talks or walks in sleep” and “trouble sleeping”. The only exception was parental ratings of overtiredness (non-CPHAUDs: 2.8%; CPHAUDs: 16.9%). CPHAUDs were more likely to be rated by their parents as overtired compared to non-CPHAUDs. Parental ratings of sleep difficulties, daytime sleepiness and sleep rhythmicity, as measured by the Pediatrics Sleep Questionnaire (PSQ) [18], showed no differences between the two groups. Another study examined whether the relationship between multiple subjective and objective sleep measures were different for CPHAUDs and non-CPHAUDs (*N* = 248; *M*_age_=10.37 (*±* 1.41); 8 to 12 years old; 48% CPHAUDs; 50% girls) at Time 1 of a longitudinal study [26]. A subset of this sample comes from the previous study. There were no significant differences on PSQ and CBCL sleep items, including overtiredness (non-CPHAUDs: 8.6%; CPHAUDs: 12.2%), though the difference was in the same direction as the previous study.

#### Self-Report

CPHAUDs and non-CPHAUDs were similar on most of the Youth Self-Report sleep items [23]. There were no significant differences on how often they reported nightmares, sleeping less or more than other children and having trouble sleeping (8 to 12 years; *N* = 248; 48% CPHAUDs; 50% girls) [27, 28]. However, CPHAUDs were less likely than non-CPHAUDs to report feeling overtired without good reason [27]. CPHAUDs reported poorer sleep hygiene than non-CPHAUDs. In particular, they were less likely to be in a good sleep environment (e.g., not listening to loud music or watching TV before going to bed) and kept a stable sleep schedule than non-CPHAUDs [29] Poor sleep environment longitudinally predicted internalizing and externalizing behaviors over time [29]. Another longitudinal study reported that parental history of alcohol use disorder had no significant relationship with the initial starting point (latent intercept) and changes of sleep difficulties and overtiredness from early to mid-adolescence (*N* = 386; 11–15 years old; 24% girls; 75% CPHAUDs) [25]. Using daily sleep diaries, one study found that CPHAUDs reported sleeping less time at night and were more likely to take naps than non-CPHAUDs (*N* = 92; 7.2–12.9 years old; 74% CPHAUDs; 37% girls) [30].

### Objective Measures of Sleep

Polysomnography (PSG) is considered the gold standard of objective sleep assessment [31–33]. PSG measures multiple sleep parameters, including brain activity (electroencephalography or EEG), heart rate, breathing and blood oxygen level. However, most polysomnographic studies occur in research or hospital sleep laboratories. The high cost and potential interruptions to participants’ daily life led researchers and clinicians to consider other assessment methods. The use of home PSG system is less costly but more research is needed to fully understand its relationship with laboratory PSG variables. The use of actigraphs, ambulatory devices that measure sleep-wake patterns via activity level, is a low-cost and non-intrusive alternative to PSG [34, 35]. It has become increasingly popular in research and clinical studies.

#### Actigraphy

In a study using actigraphy to measure sleep, children wore an actigraph watch for seven days. CPHAUDs showed significantly shorter total sleep time (TST) (18 min) and had more nighttime motor activity than non-CPHAUDs, (7.2–12.9 years old; *N* = 92; 74% CPHAUDs; 37% girls) [30]. Another study using a subset of the subjects in [30] reported shorter TST among CPHAUDs compared with non-CPHAUDs (7.2–13.0 years old; *N* = 82; 79% CPHAUDs; 35% girls) [36]. CPHAUDs also went to bed later and spent less time in bed on weekends [36].

A three-year longitudinal study reported no significant differences between the two groups on total sleep time (TST), sleep onset latency (SOL), sleep efficiency (SE) and wake time after sleep onset (WASO) at Time 1 (T1) (8 to 12 years; *N* = 248; 48% CPHAUDs; 50% girls) [27, 28]. CPHAUDs had significantly shorter TST than non-CPHAUDs one year later (T2) [27]. The average difference was small (about 9 min). CPHAUDs also had significantly shorter WASO than non-CPHAUDs. This may be affected by the shorter TST among CPHAUDs. The percentage of wake time after sleep onset is largely the same across the two groups (12% among CPHAUDs and 11% among non-CPHAUDs).

#### Polysomnography (PSG)

A cross-sectional study examined sleep EEG among CPHAUDs and non-CPHAUDs with no known psychiatric or substance use problems. Results indicated that CPHAUDs and non-CPHAUDs were not different on any sleep stage variables, such as minutes of Stages 1, 2, slow wave sleep (SWS), rapid eye movement (REM) latency, SOL, TST, time in bed or WASO (9–10 years old, *N* = 30; 43% CPHAUDs; 37% girls) [37]. There were no signs of sleep disruption in any sleep stages in both groups. However, CPHAUDs showed less normalized power in the delta band and spindle range during NREM sleep than non-CPHAUDs [37].

A longitudinal study assessed a child sample (9–10 years old, *N* = 24; 42% CPHAUDs; 33% girls) and a teen sample (15–16 years old, *N* = 25; 40% CPHAUDs; 64% girls) twice, approximately 1.5 to 3 years apart [38]. A subset of the child sample also participated in the cross-sectional study described in the previous paragraph. Consistent with the cross-sectional study, there were no difference in any sleep stage variables in either sample. In the child sample, compared to non-CPHAUDs, CPHAUDs exhibited lower normalized EEG power in the delta band for the left occipital derivation during NREM sleep in the initial assessment. In the teen sample, there were no differences between the two groups in normalized power for any frequency or any sleep state. A comparison of absolute power showed differences among CPHAUDs and non-CPHAUDs. In the child sample, the differences were found in the initial assessment only. During NREM sleep, CPHAUDs had lower EEG power than non-CPHAUDs in all frequency bands in the right occipital derivation. During REM sleep, CPHAUDs had lower EEG power in the delta and sigma band than non-CPHAUDs in the right occipital derivation. In contrast, CPHAUDs exhibited greater power than non-CPHAUDs for the theta and alpha bands during REM sleep in the right central derivation. In the teen sample, CPHAUDs exhibited less absolute power than non-CPHAUDs in the delta band for the central derivations during NREM sleep at both assessments. During REM sleep, CPHAUDs exhibited less delta band power than non-CPHAUDs in the left central derivation in the initial assessment and in the right central derivation during the follow-up assessment.

In a recent study that consisted of a larger sample of children (8 to 12 years; *N* = 248; 48% CPHAUDs; 50% girls), there were no significant differences on any sleep stage variables with one exception. CPHAUDs reported significantly longer sleep onset latency than non-CPHAUDs, though the difference was small (about 8 min) [27]. The analysis on spectral power differences between the two groups is still ongoing and therefore cannot be presented in this paper.

Last but not least, in an earlier cross-sectional study, sleep EEG of 18 depressed adolescents who had a parental history of alcohol use disorder were compared to 14 depressed adolescents without such history (8 to 16 years; *N =* 32; 56% CPHAUDs; 53% girls) [39]. All participants were naïve to alcohol use at the time of the study. There were no differences in sleep stage variables between the two groups. However, there was a significant COA x sex interaction. Male CPHAUDs had greater alpha band power in NREM and REM sleep than male non-CPHAUDs. In contrast, there were no such differences between female CPHAUDs and non-CPHAUDs. This finding indicated that CPHAUDs and non-CPHAUDs might differ in spectral power during sleep. As depression has been shown to affect alpha band power, it is unclear whether the findings were influenced by participants’ depression, parental history of AUD or both.

## Parental Problem Drinking and Offspring Sleep Characteristics

Several studies have examined the relationship between parental problem drinking and children’s sleep. These studies recruited children of parents who have different alcohol-related problems (definitions provided below) but did not formally interview parents via a structured diagnostic interview and therefore parental AUD diagnosis could not be determined. In a cross-sectional study, parents reported their own alcohol-related problems (e.g., “got into trouble with the law while drinking”, “drinking resulted in an argument/fight with family members” and “got into a fight or hearted argument with a stranger while drinking”) on the Parental Alcohol Experiences Scale (PAES) [40]. The PAES consists of 15 items that assess AUD symptoms across a range of family, social, legal, and work/school contexts. A small percentage of these parents exceeded the cut-off score of PAES, which potentially indicate the presence of an alcohol use disorder (3% women, 12% men). Children wore an actigraph watch for seven nights (*N* = 282; Mean age = 9.44 (SD = 8.55); 8–12 years old; 48% girls; 35% Black) [41]. Children’s ethnicity and socioeconomic status (SES) was used as moderators in the analyses. Controlling for important covariates that were known to correlate with parental problem drinking and children’s sleep, parental problem drinking was associated with shorter sleep duration, reduced sleep efficiency and greater long wake episodes among Black children and participants with lower SES.

A follow-up study of the same sample was conducted one year later [42]. Longitudinal data indicated that there were reciprocal relations between parental problem drinking and children’s sleep. After controlling for autoregressive effects (relations between constructs over time) and important covariates, father’s problem drinking predicted reduced sleep duration and efficiency of children over time. More frequent long wake episodes among children longitudinally predicted greater parental problem drinking. SES significantly moderated the relationship between father’s problem drinking and four actigraphy sleep parameters in children. Father’s problem drinking predicted shorter sleep duration, lower sleep efficiency, more frequent long wake episodes and longer sleep onset latency among children with lower SES. However, the relationship was not significant among children with higher SES.

In a large population study of two-parent families, the relationship between parental drinking, mental health and education was used to predict offspring’s subsequent prescriptions drugs for sleep and mental health problems [43]. Two different risk profiles increased the risk of being dispensed prescriptions drugs for sleep and anxiety/depression later: (i) low education, symptoms of mental health problems and weekly binge drinking in both parents and (ii) frequent heavy drinking in both parents and symptoms of mental health problems in father. This longitudinal study provides evidence that parental problem drinking predicts subsequent sleep problems in their offspring.

## Family History of Alcohol or Substance Use and Offspring Sleep

We examined research on the relationship between a family history of alcohol/substance use on offspring’s sleep as it may shed light on how parent’s AUD may affect the sleep of their children. There are only a few studies in this area. We selected two that are most relevant to the objective of this review. Instead of focusing on parental history of AUD, these two studies used broader definitions, including first or second degree of biological relatives or the AUD history of one’s grandparents. The specific definitions used in each study were described below.

After alcohol intoxication or a placebo, those with and without a family history of alcohol use disorder (at least one first or second degree biological relative) did not differ on objective sleep measures (total sleep time, sleep efficiency, nighttime awakening, wake after sleep onset) [44]. However, after the alcohol/placebo manipulation, those with a family history of alcoholism self-reported less morning sleepiness and better sleep quality the prior night compared to those without a family history of alcoholism. Given the small number of men in their study with a family history of alcoholism [44], these results need to be interpreted with caution.

Youth with a family history of alcohol or substance use problems (at least one parent or two grandparents had lifetime AUD or SUD symptoms) had greater parental reports of sleep disturbances than those without a family history of alcohol or substance use problems [45]. Youth with a family history of alcohol or substance problems also showed greater cortical thinning than those without such history. Sleep disturbances were associated with cortical thinning [45]. Although the authors did not directly test the mediating effect of cortical thinning on the relationship between family history of alcohol/substance use problems and sleep outcomes, their analyses highlight the biological impacts of this family history and how physical changes in the brain may impact sleep outcomes.

## Summary of Current Research Findings

Previous research established a longitudinal relationship between sleep disturbances (e.g., having trouble sleeping, irregular sleep schedules, insufficient sleep and low sleep quality) with early onset of substance use and related problems among both CPHAUDs and non-CPHAUDs [24, 25, 46]. CPHAUDs are at risk for developing alcohol- and substance-related problems [3, 8]. They are also more likely to develop an AUD and an SUD [3, 6, 7]. Current research appears to show inconsistent findings on sleep measures of the two groups. Studies using parental ratings and self-report show that the two groups are largely similar to one another [24–26]. One study found that CPHAUDs were more likely to be rated by parents as overtired for no particular reasons than non-CPHAUDs [26]. However, this finding was not replicated when more subjects were recruited, though the difference was in the same direction [28]. CPHAUDs were more likely than non-CPHAUDs to report feeling less tired [27, 28]. They were also more likely than non-CPHAUDs to report having a poorer sleep environment and a less stable sleep schedule [27]. A poor sleep environment was correlated with both internalizing and externalizing problems over time [29].

Actigraphy data showed that that CPHAUDs were significantly more likely to sleep less than non-CPHAUDs, though the differences were small [27, 30, 36]. PSG data suggested that CPHAUDs were more likely to have less power in the delta and sigma bands [37, 38]. The authors stated that the findings were consistent with several studies comparing alcohol-dependent and abstinent AUD individuals and individuals without AUD. In those studies, AUD individuals showed reduced spectral power in the delta and theta bands during NREM sleep compared with non-AUD individuals [47–49]. Another study using PSG data found that CPHAUDs reported a slightly longer sleep onset latency compared with non-CPHAUDs [27]. Taken as a whole, existing research shows that CPHAUDs and non-CPHAUDs are similar on most objective and subjective sleep measures. There are several significant differences between the two groups (e.g., poor sleep hygiene) that may have implications for the development of behavioral problems, substance use and other risk behaviors.

## Unanswered Questions and Future Directions

There are many unanswered questions in this area that future research needs to address. We discussed a few of those questions here. The effects of parental AUD on offspring development as a whole and sleep in particular is a complex issue. Current research used different definitions of parental AUD history. Most existing studies assessed lifetime AUD [24–26, 30, 36–38, 46] while some examined problem alcohol use with only a small percentage of participants that met AUD criteria [41, 42]. In studies that assessed parental AUD, some used one assessment method, e.g., a structured diagnostic interview [37, 38], while others used both structured diagnostic interviews and other information, e.g., court record [24, 25, 30, 36, 46] and the Michigan Alcohol Assessment Test [26–29]. The inconsistency in findings in these studies may stem in part from different definitions and assessments of parental AUD. Future studies that use multiple assessment methods to examine parental AUD may help to clarify what specific aspects of parental AUD history are most predictive of different sleep variables. Additionally, future research could control for when the lifetime parental AUD was diagnosed. More recent parental AUD diagnoses (e.g., past year or diagnoses obtained subsequent to the child’s birth) will likely impact children’s sleep more than parental diagnoses in the distant past.

Demographic variables such as race, ethnicity, socioeconomic status (SES) and sex need to be accounted for when we interpret the findings presented in this paper. Some studies used only White participants [24, 25, 46]. Others include a sizable proportion of Black and Hispanic participants [36, 41, 42]. Even though these studies controlled for race/ethnicity in their findings, the issue of whether race and ethnicity may interact with parental AUD to affect sleep was explored in some studies only [41, 42]. SES might affect parental availability and children’s immediate home environment, which could in turn affect children’s sleep and behaviors. SES may also interact with sleep to affect behaviors. Most studies reviewed in this paper reported the presence or absence of sex differences on sleep measures and controlled for the effects of sex when analyzing the relationship between sleep and behavioral outcomes. However, none of these studies examined whether sex interacted with sleep measures to affect behaviors. The effects of race, ethnicity, SES and sex on children’s sleep may be independent of parental AUD. Or they may interact with parental AUD to affect children’s sleep. These are issues that future research could address.

Most of the studies reviewed in this paper examined offspring’s sleep at an early age, from early childhood to mid-adolescence (see Table 1). Only a few studies examined sleep variables among CPHAUDs and non-CPHAUDs in late adolescence and emerging adulthood [38, 43, 44, 46]. There are remarkable changes in sleep from infancy to adulthood, e.g., sleep duration, sleep regularity, sleep timing, sleep architecture, slow-wave sleep characteristics and their topography etc [50, 51]. Changes in slow wave activity (SWA) are associated with cortical maturation and synaptic pruning [50]. For instance, decreased synaptic density following pruning is associated with lower SWA amplitude [52, 53]. The locations where maximal SWA can be observed switch from the posterior to anterior brain regions from childhood to adolescence, which parallels the time course of cortical maturation [54–56]. According to the two-process model of sleep regulation, sleep duration and timing are regulated by both the homeostatic sleep-wake process (Process S) and circadian rhythm (Process C) [57–59]. Both processes undergo dramatic changes in adolescence [60] and emerging adulthood [51]. These changes interact with academic requirements (e.g., school work in high school and college), social factors (e.g., extracurricular activities, time spent with friends and social media) and increases in freedom among adolescents and emerging adults to determine the amount and timing of their own sleep [51, 60]. The differences in sleep characteristics in CPHAUDs compared with non-CPHAUDs earlier in life (e.g., lower spectral power of delta waves [37, 38], shorter total sleep duration [27, 30], worse sleep hygiene practices [27]) may change over time. It is therefore important for future studies to include participants from older age groups, particularly mid- and late adolescence and emerging adulthood, when the brain continues to mature. It is also important to follow-up with children and adolescents for longer periods of time (e.g., from early childhood to late adolescence, from early adolescence to emerging adulthood) so that developmental differences in age and behavioral outcomes can be examined.

Another future direction that deserves attention is the bidirectional relationship between sleep and alcohol/substance use [61]. The relationships between sleep and alcohol/substance use are likely reciprocal and dynamic (ever-changing). COA may be an important moderator of these relationships. To our knowledge, no extant study has directly examined whether and how CPHAUDs and non-CPHAUDs may be different in the sleep-alcohol relationship across multiple developmental periods. Future studies, especially those that employ intensive longitudinal methods (e.g., ecological momentary assessment, daily diaries) and multiple assessment of sleep (e.g., combining subjective report with actigraphy and PSG data) are particularly promising.

### Implications for Prevention, Intervention and Treatment

Current research indicates that CPHAUDs and non-CPHAUDs are similar on most sleep measures. Sleep disturbances appear to be a general risk factor for alcohol and substance use for all individuals, regardless of their parents’ alcohol use. It is therefore useful to address both sleep problems and sleep health [62] in prevention, intervention and treatment programs for substance use. Participants of these programs who report sleep problems could receive information, advice, guidance and treatment for their sleep disturbances. Participants without sleep problems and their parents/families could receive preventative information about sleep health, i.e., sleep duration, sleep continuity/efficiency, timing, daytime alertness/sleepiness and satisfaction/quality [62] and sleep hygiene. Program directors and health care providers might want to initiate a discussion with participants and their families on these topics and provide suggestions on how to promote sleep health and good sleep hygiene. All participants, whether they have sleep disturbances or not, could be encouraged to discuss potential obstacles for implementing these suggestions.

Existing research indicates that some CPHAUDs appear to have shorter sleep duration [27, 30, 36] and poorer sleep hygiene compared with non-CPHAUDs between the ages of 8–12 [27]. PSG studies show that CPHAUDs have lower spectral power in the delta frequency band than non-CPHAUDs [37, 38], a finding that is consistent with studies comparing alcohol-dependent adults and AUD individuals who abstain from alcohol. One recent study indicates that CPHAUDs appear to have slightly longer sleep onset latency than CPHAUDs, as measured by PSG in a hospital sleep lab [27]. These PSG findings need to be replicated in future research. It is unclear whether these differences will continue or change as CPHAUDs get older. If such differences continue and increase overtime, they may interact with one another (e.g., poorer sleep hygiene and an inconsistent sleep schedule may further reduce total sleep duration) or with other risk factors (e.g., low parental monitoring, poverty). Additionally, more studies on cortical thinning and differences in brain anatomy due to family history of substance use could be conducted as these differences might indicate important biological mechanisms contributing to sleep difficulties in these populations. Programs designed to prevent alcohol and substance misuse among CPHAUDs could share this information with their participants. The importance of sleep hygiene and sleep health could be discussed. The information may be especially useful for intervention and treatment programs designed for CPHAUDs who report alcohol use/alcohol-related problems and concomitant sleep disturbances. These disturbances could be addressed and when appropriate, should be treated promptly without delay.

## Conclusion

The research findings reported in this review paper indicate that CPHAUDs and non-CPHAUDs are similar on most sleep characteristics. As pointed out earlier in the paper, there are still many unanswered questions. More work needs to be done to address these questions and replicate the findings of extant research. In particular, it is important for future studies to specify the nature of parental problem drinking and diagnosis, use multiple assessment methods, differentiate between more distant and recent parental AUD diagnoses and control for parental current drinking patterns. It is essential for researchers to diversify the COA samples to include individuals of different age groups, SES, and race/ethnicity. At the minimum, demographic variables that may affect parental availability, motivation and monitoring need to be controlled for when examining the relationship between parental history of AUD and children’s sleep. Studies that use a longitudinal design, especially those that follow CPHAUDs from childhood to mid- and late adolescence and emerging adulthood using both objective and subject sleep measures, will elucidate how COA status affects changes in sleep over time. The development of sleep (including duration, timing and consistency) is affected by the complex interplay between individual level factors (e.g., biological factors such as puberty, changes in cognitive processes, social relationships), family dynamics (e.g., parental support, guidance, monitoring, awareness of child sleep-wake schedule, conflict between parents), out-of-home influences (e.g., school, extracurricular activities, employment, church) and sociocultural factors (e.g., SES, poverty, cultural values on work and rest) [41, 42, 51, 60]. Understanding how parental history of AUD may interact with these factors to affect children’s sleep may lead to new insights and help resolve existing inconsistencies in research findings.

## Data Availability

No datasets were generated or analysed during the current study.
